# Recent developments in podiatric prescribing in the UK and Australia

**DOI:** 10.1186/1757-1146-2-37

**Published:** 2009-12-15

**Authors:** Mark F Gilheany, Alan M Borthwick

**Affiliations:** 1 Podiatrists Registration Board of Victoria, Melbourne, Victoria, Australia; 2 School of Health Sciences, University of Southampton, UK

## Abstract

Recent and substantial changes in access to restricted medicines by podiatrists in Australia are clearly consistent with healthcare policies aimed at reshaping the healthcare workforce. At the same time, prescribing and access to medicines by allied health professionals, including podiatrists, has been the focus of a recent scoping project by the UK Department of Health. In this commentary we explore the possible implications of these changes.

## Background

Non-medical prescribing has been viewed as a challenging transition in professional role boundaries, as well as a necessary component of workforce redesign essential to the creation of a sustainable health service [1-3]. There is little doubt that the need for non-medical healthcare professionals to assume new roles, including those previously exclusive to the medical profession, constitutes part of the drive towards long-term sustainability and affordability in health care provision across the Western world. In such a climate, change may be inevitable, but it has certainly not been effortless. As Britten [4] has pointed out, prescribing remains "one of the core activities that demarcate the medical profession from other groups...", indicating the extent to which workforce 'flexibility' impacts on 'traditional' role boundaries.

Clearly, non-medical prescribing has emerged as a result of healthcare policies seeking to address pressing demographic and economic concerns [5,6], and these imperatives continue to drive forward the 'extended scope' agenda. Podiatric prescribing is one such example, as well as an exemplar illustrating the difficulties posed in transferring role responsibilities from one profession to another [2]. Understanding the contemporary context of these changes is dependent upon an appreciation of the complex socio-historical developments which preceded them, and the paper by Borthwick et al, recently published in Journal of Foot and Ankle Research, may be used as a yardstick for judging the progress made over many years [7]. In this commentary, however, the authors focus on two of the most recent events, and consider what these may mean for future practice.

## Recent developments in the UK

In July of 2009 the UK Department of Health published a report on the recent scoping project undertaken to re-examine the case for enhanced access rights to medicines by the allied health professions [8]. Whilst the focus of the study was to reconsider the utility and applicability of all existing mechanisms for accessing restricted category medicines, the final recommendations are worthy of comment, because they assert that there is a 'strong case for progression to independent prescribing' by podiatrists and physiotherapists [8]. It also suggests further funded exploratory research to inform how these key recommendations can be taken forward. It may even be fair to suggest that it is an indication of the extent to which the Department of Health now considers the prescribing of medicines by allied health professionals to be a safe and effective use of resources in answer to the growing needs of patients and their health service providers, both in terms of responsiveness and adaptability.

The challenge for the profession of podiatry will be to ensure that sufficient numbers of practitioners undertake the additional training and education necessary to carry out these tasks, and to ensure that these skills are fully utilised in practice. Although podiatric surgeons already possess the necessary training and skills, and would undoubtedly benefit from early recognition as independent prescribers, other services would also benefit from this enhanced scope - such as podiatrists specialising in diabetic foot care, where, for example, a rapid response to foot infections is critical [9]. As 'supplementary' prescribers, many already do. Yet practitioners working in general practice should not view themselves as excluded from these roles, and must also consider the contribution that they can make to ensuring the provision of healthcare fit for purpose in the 21^st ^century.

Clearly, the Department of Health has taken another significant step, reflecting the current health policy direction and a recognition of the advances in allied health clinical practice. Policy development leading to further regulatory change and eventual implementation is, however, likely to be a slow process, if the Australian experience is to be considered a guide.

## Recent developments in Australia

The extension of prescribing rights for non-medical practitioners in Australia has been problematic; in part due to the structural complexity of the Australian health care system. Whereas the UK health professions have one registration authority, providing a uniform approach to regulation, Australia has eight states and territories, each with separate legislation for both professional and 'poisons' regulation. This is complicated further by current funding arrangements, which operate on a complex public and private system model in which funding for services provided by medical practitioners take precedence and services provided by non medical practitioners is limited. A further barrier to access is the cost of restricted medicines which are subsidised under the Pharmaceutical Benefits Scheme (PBS). The PBS does not automatically extend to non medical prescribers. It is not, then, merely a question of regulation - it is inclusion within the PBS that is necessary if patients are to be treated equally. The position of podiatric surgery in Australia reflects the impact that inequity with funding can have. Australian podiatric surgeons (the first podiatrists to gain prescribing rights in Australia) face significant barriers to providing a full contribution to the health workforce [10]. This is despite broad recognition of the need for role flexibility [11-13].

Against the backdrop of these structural difficulties, the Victorian podiatry profession (approximately 1/3 of the podiatric profession in Australia) was recently granted an extension of scope of practice to include prescription of restricted medicines. The Victorian legislation (Health Practitioners Act 2005) acknowledges podiatrists as prescribers of restricted substances. Implementation is progressing such that all graduate podiatrists are now able to be endorsed to prescribe (after completion of the endorsement process) a broad range of clinically appropriate restricted medicines. The first podiatrists with these rights are expected to be endorsed by the Podiatrists Registration Board of Victoria by the end of 2009.

The imminent arrival of a new national board for all health professionals, in July 2010, promises to ensure a high degree of uniformity (at least in principle) [14]. Initially within this scheme (as far as medicines are concerned) individual state poisons regulations will still apply, which will delay Australia-wide application of the Victorian reform agenda. Indeed, it is yet to be determined whether the Victorian model will be adopted as a National framework by the new National Podiatry Board.

## Discussion

In Victoria, the co-operative approach to reform demonstrated by the regulatory body (registration board), educational institutions, professional bodies and government departments has demonstrated what can be achieved. The process, however, took over 15 years, involving extensive stakeholder engagement and curriculum reform.

The result is that Victorian podiatrists are now provided, at a graduate level, with a sufficient grounding in the medical sciences to register as health practitioners able to prescribe restricted medicines.

The reform in Victoria sets a new benchmark for Australian podiatric education and scope of practice. There are significant long term and broader implications for the position of podiatry in the Australian health sector. It is acknowledged that there is sufficient clinical need and appropriate educational background to enable the prescribing of restricted pharmacological agents by podiatrists. Importantly, this acknowledgment is not confined to particular specialist areas of practice (such as surgery) - which represents a paradigm shift.

Given the timeframe for reform and implementation observed in Victoria, it will be of interest to monitor the speed of further reform in the UK. It is intriguing to consider how "a strong case for independent prescribing" might be translated into practice and if it will resemble the developments in Victoria. In Australia, ongoing interest will relate to how the Victorian model will affect the national scene, but with the emergence of a national registration board it is possible that this approach will be endorsed by a national podiatry board, and that local state jurisdictions will seek to amend their poisons regulations accordingly. Funding imbalances are likely to be addressed only when a uniform and National approach to prescribing is in place. For those interested in how the new rights should work in practice, it would be worth viewing the website of the Podiatrists Registration Board of Victoria, where a section is devoted to the recent S4 issue [15].

In summary, non-medical prescribing is a pragmatic and workable solution to a major challenge facing health services across the Western world. Even now it appears to be proving its worth, increasing the rate at which health care practitioners are utilised for skill sets rather than governed by lines of demarcation.

## Competing interests

One author (AMB) is currently Deputy Editor (UK) of the Journal of Foot and Ankle Research, and first author of a paper referred to directly in this commentary.

## Authors' contributions

Both authors were equally involved in the design and writing of the paper. AMB initially drafted the overall context and the UK element of the manuscript, and MFG drafted the Australian context, with additions to the context. Critical revision was undertaken by both authors. Both authors contributed to the interpretation offered.
